# Supplementary material for: Analysis of Volatile Profile and Aromatic Characteristics of Raw Pu-erh Tea during Storage Based on GC-MS and Odor Activity Value
Source: Foods. 2023 Sep 26;12(19):3568. doi: 10.3390/foods12193568 (PMC10572200; doi:10.3390/foods12193568)
Supplement: Supplementary file 1 [file foods-12-03568-s001.zip › foods-2606523-supplementary.pdf]

Supplementary Materials

Table S1

Volatile compounds identified in RAPT samples from different storage durations

Volatile compounds	RI	Concentration (μg/100g)										
		T0	T1	T2	T3	T4	T5	T6	T7	T8	T9	T10
Hexanal	800	−	0.45±0.06	6.04±0.80	−	−	1.38±0.22	0.41±0.02	0.52±0.06	0.53±0.01	0.64±0.12	0.65±0.05
3−methyl−Butanoic acid	811	4.44±0.50^{ef}	5.86±1.09^{g}	1.92±0.03^{bc}	3.04±0.48^{d}	5.12±0.40^{f}	3.75±0.37^{de}	0.66±0.06^{a}	1.81±0.28^{bc}	1.22±0.06^{ab}	1.27±0.10^{ab}	2.08±0.03^{c}
2−methyl−Butanoic acid	811	5.03±0.82 ^{f}	4.55±0.76 ^{f}	2.37±0.03 ^{c}	3.26±0.32 ^{e}	3.13±0.05 ^{de}	2.52±0.31 ^{cd}	0.61±0.10 ^{a}	1.09±0.19 ^{ab}	0.64±0.13 ^{a}	1.35±0.23 ^{b}	1.43±0.08 ^{b}
1−ethyl−1H−Pyrrole	829	1.37±0.02	0.95±0.03	−	−	−	−	−	−	−	−	−
1,2,5,5−tetramethyl−1,3−Cyclopentadiene	840	2.78±0.36^{bc}	2.32±0.07^{bc}	1.44±0.26^{a}	5.37±0.76^{e}	3.68±0.63^{d}	2.02±0.25^{ab}	2.26±0.11^{bc}	1.52±0.23^{a}	2.38±0.26^{bc}	2.2±0.140^{bc}	4.30±0.33^{d}
(Z)−3−Hexen−1−ol	851	3.02±0.21	2.92±0.28	−	−	−	−	−	−	−	−	−
2−Hexenal	853	−	−	3.34±0.56	6.65±0.39	11.02±0.49	3.75±0.44	5.88±1.00	3.16±0.53	7.68±1.61	6.13±0.51	6.79±0.82
1−Hexanol	867	5.58±0.07^{f}	5.80±0.79^{f}	3.05±0.03cd	4.06±0.30^{e}	4.62±0.73^{e}	3.20±0.39^{d}	2.29±0.10^{b}	2.14±0.36^{ab}	3.01±0.16^{cd}	1.53±0.31^{a}	2.36±0.35^{bc}
p−Xylene	870	1.79±0.28^{ab}	1.92±0.21^{abc}	1.72±0.29^{ab}	5.71±0.40^{f}	4.22±0.61^{e}	1.67±0.32^{a}	2.62±0.07^{d}	2.54±0.26^{d}	2.88±0.03^{d}	2.46±0.18^{cd}	2.28±0.46^{bcd}

2−Heptanone	889	4.15±0.54^{de}	3.94±0.20^{de}	2.78±0.01^{ab}	3.81±0.57^{c}	5.83±0.77^{f}	4.58±0.08^{e}	2.99±0.29^{ab}	2.36±0.30^{a}	3.06±0.11^{b}	3.98±0.12^{de}	2.34±0.17^{a}
Heptanal	901	4.58±0.76^{ab}	4.51±0.48^{ab}	7.8±0.18^{de}	6.68±0.53^{cd}	8.71±1.07^{e}	7.09±0.42^{d}	7.03±1.17^{d}	3.42±0.45^{a}	7.60±0.13^{de}	5.69±0.35^{bc}	5.16±1.03^{b}
2−Propenoic acid, butyl ester	902	1.23±0.13	−	−	−	−	−	−	−	−	−	−
2−butoxy−Ethanol	907	−	0.46±0.05	−	−	1.59±0.02	−	0.94±0.18	0.50±0.06	0.38±0.07	0.64±0.07	0.66±0.09
Hexanoic acid, methyl ester	924	−	−	−	−	0.99±0.06	−	0.39±0.04	2.36±0.30	0.50±0.03	−	−
Camphene	933	−	−	−	−	−	−	1.20±0.10	1.32±0.23	−	−	−
α−Pinene	939	0.58±0.09^{ab}	0.64±0.07^{bc}	0.45±0.07^{a}	0.50±0.01^{a}	0.78±0.03^{cd}	0.77±0.11^{cd}	0.82±0.12^{d}	1.05±0.09^{e}	1.04±0.07^{e}	1.04±0.08^{e}	0.75±0.04^{cd}
2−Octanone	952	0.53±0.04	−	−	−	−	1.36±0.24	−	−	−	−	−
Hexanoic acid	961	26.59±3.60	25.95±2.40	9.51±1.53	−	27.39±0.27	26.98±5.34	−	−	−	−	−
1−(3−methylenecyclopentyl)−Ethanone	963	−	−	−	−	−	−	0.73±0.03	0.59±0.09	−	−	−
Benzaldehyde	966	14.77±0.67^{a}	18.15±0.58^{ab}	35.90±4.64^{d}	26.23±1.89^{c}	46.78±4.13^{e}	24.25±4.58^{bc}	18.65±1.86^{ab}	20.73±1.42^{abc}	23.83±4.25^{bc}	21.93±4.13^{bc}	24.15±4.26^{bc}
3,5,5−trimethyl−2−Hexene	968	37.93±1.16^{g}	19.55±2.69^{f}	7.66±1.02^{d}	7.12±0.70^{cd}	10.46±1.57^{e}	8.69±1.55^{de}	3.33±0.50^{ab}	3.30±0.58^{ab}	2.89±0.56^{a}	4.41±0.10^{ab}	5.28±0.87^{bc}

2-Methylenecyclohexanol	974	–	–	–	–	–	–	2.71±0.49	3.34±0.68	3.81±0.08	4.78±0.69	–
1-Octen-3-ol	976	95.72±2.24^{g}	51.67±5.98^{f}	26.59±2.40^{d}	23.11±2.95^{cd}	36.69±1.59^{e}	21.79±1.34^{c}	11.90±0.74^{a}	12.06±2.07^{a}	12.57±1.39^{ab}	17.11±0.51^{b}	15.51±2.53^{ab}
2,3-Octanedione	986	7.45±0.87^{c}	4.24±0.16^{ab}	5.39±0.86^{b}	9.51±1.89^{d}	7.45±1.40^{c}	4.07±0.83^{ab}	3.12±0.16^{a}	2.74±0.52^{a}	3.71±0.43^{a}	2.65±0.02^{a}	3.08±0.32^{a}
6-methyl-5-Hepten-2-one	987	19.46±1.55^{ef}	14.85±0.99^{cd}	12.83±1.47^{abc}	16.39±3.17^{cde}	21.93±3.56^{f}	17.33±3.32^{de}	10.80±1.05^{ab}	9.99±1.93^{ab}	9.30±1.33^{a}	13.45±0.59^{bc}	12.93±0.69^{abc}
β-Myrcene	992	–	17.95±0.84	–	–	–	21.02±3.18	–	–	–	16.13±0.84	12.85±1.02
(2R,5R)-2-Methyl-5-(prop-1-en-2-yl)-2-vinyltetrahydrofuran	993.8	4.74±0.32^{a}	5.62±0.07^{ab}	4.89±0.76^{ab}	7.50±1.42^{cd}	11.66±1.55^{e}	6.53±1.16^{abc}	5.03±0.01^{ab}	5.78±1.14^{abc}	6.62±0.14^{bc}	8.92±1.46^{d}	6.30±0.58^{abc}
Octanal	1001	7.28±0.09^{ab}	6.62±0.67^{ab}	6.01±0.27^{a}	6.26±1.19^{a}	18.80±0.89^{d}	11.07±1.91^{c}	6.98±0.59^{ab}	6.21±0.97^{a}	8.56±1.28^{b}	8.36±1.42^{b}	7.43±1.07^{ab}
(E,E)-2,4-Heptadienal	1011	11.52±0.35^{abc}	10.40±0.30^{a}	10.68±2.06^{ab}	15.28±0.40^{e}	18.85±1.86^{f}	11.11±1.87^{ab}	13.18±1.21^{bcde}	12.34±2.32^{abcd}	14.41±0.18^{de}	14.13±0.28^{de}	13.92±1.45^{cde}
α-Terpinene	1018	4.93±0.23^{ab}	5.96±0.12^{cd}	4.64±0.50^{ab}	6.40±0.19^{d}	8.56±0.11^{e}	5.97±0.39^{cd}	4.82±0.46^{ab}	5.12±0.45^{bc}	5.40±0.35^{bc}	6.67±1.18^{d}	4.03±0.64^{a}
Benzyl chloride	1019	1.50±0.05^{d}	1.01±0.13^{abc}	1.06±0.18^{bc}	1.62±0.08^{de}	1.72±0.07^{e}	1.05±0.18^{abc}	1.15±0.15^{bc}	0.97±0.16^{ab}	1.22±0.09^{c}	0.82±0.09^{a}	0.94±0.01^{ab}
o-Cymene	1026	4.22±0.16	–	–	15.27±2.08	–	19.3±3.55	–	–	–	–	–

2−ethyl−1−Hexanol	1026.3	3.74±0.64^{a}	56.79±4.80^{f}	33.71±6.19^{c}	32.19±3.17^{c}	60.63±5.05^{f}	19.3±3.55^{b}	28.46±0.87^{c}	49.97±1.65^{e}	42.02±0.52^{d}	56.39±0.84^{f}	49.57±3.43^{e}
D−Limonene	1030	44.37±7.79^{b}	68.31±1.50^{d}	74.91±12.27^{cd}	27.88±3.64^{d}	74.48±4.30^{a}	57.35±11.33^{bc}	45.45±3.18^{b}	55.43±8.92^{b}	53.03±4.01^{b}	69.44±5.84^{cd}	43.89±7.40^{b}
Benzyl alcohol	1034.4	14.21±0.19	−	−	12.43±0.26	−	−	−	−	−	−	−
2,6−dimethyl−5−Heptenal	1044	25.51±0.37^{e}	21.35±1.30^{cd}	18.09±3.51^{bc}	25.65±2.28^{e}	24.54±3.10^{de}	20.66±2.77^{c}	13.24±0.91^{a}	14.65±2.43^{ab}	15.89±0.06^{ab}	13.44±1.49^{a}	12.94±0.81^{a}
Benzeneacetaldehyde	1049	11.93±0.26^{ab}	9.98±0.62^{a}	11.99±1.36^{ab}	22.67±0.96^{d}	22.44±3.00^{d}	11.63±1.93^{ab}	12.24±1.19^{ab}	12.24±2.10^{ab}	19.25±0.65^{c}	14.11±0.30^{b}	17.16±0.73^{c}
(Z)−3,7−dimethyl−1,3,6−Octatriene	1051	6.53±0.26^{ab}	8.29±0.68^{cde}	7.43±0.68^{abcd}	9.44±0.35^{e}	10.86±0.37^{f}	7.81±0.93^{bcd}	6.94±0.38^{ab}	6.44±0.86^{a}	7.14±0.44^{abc}	8.52±0.54^{de}	7.54±1.29^{abcd}
1H−Pyrrole−2−carboxaldehyde, 1−ethyl−	1054	25.94±0.51^{e}	23.39±2.05^{de}	23.00±4.15^{de}	24.66±3.13^{de}	32.01±2.66^{f}	20.12±3.50^{cd}	10.05±0.31^{a}	12.15±2.43^{a}	9.05±1.36^{a}	13.20±0.29^{ab}	17.39±3.04^{bc}
2−Octenal, (E)−	1056.7	−	−	−	−	−	−	−	2.11±0.31	−	2.87±0.05	−
Ethanone, 1−(1H−pyrrol−2−yl)−	1063.2	13.99±1.34^{bc}	13.35±0.74^{b}	13.25±0.65^{b}	25.82±0.14^{d}	23.12±2.73^{d}	16.67±2.63^{c}	11.84±0.82^{ab}	12.01±1.22^{ab}	12.96±2.29^{ab}	12.39±2.09^{ab}	9.93±1.76^{a}
γ−Terpinene	1064	12.38±2.06^{bc}	13.80±1.93^{cd}	15.40±2.70^{d}	12.75±0.50^{bcd}	10.23±0.88^{b}	6.90±0.21^{a}	6.32±0.08^{a}	10.18±1.49^{b}	7.18±0.67^{a}	13.09±0.20^{cd}	12.61±2.44^{bcd}
(E)−2−Octen−1−ol	1067	59.44±2.59^{g}	34.28±3.24^{f}	28.8±2.29^{e}	27.26±0.19^{e}	27.93±0.66^{e}	18.67±1.05^{d}	13.39±0.38^{c}	9.72±1.11^{b}	6.22±0.82^{a}	9.99±0.55^{b}	9.81±0.55^{b}

1−Octanol	1068	20.41±0.43c	25.39±1.53^{d}	27.68±5.06^{d}	49.66±0.06^{f}	39.84±2.93^{e}	26.91±4.28^{d}	8.53±1.21^{ab}	7.62±0.37^{a}	12.67±0.51^{b}	10.15±0.95^{ab}	10.55±1.29^{ab}
trans−Linalool oxide (furanoid)	1076	46.04±4.44^{ab}	44.25±4.77^{ab}	61.16±6.85^{cd}	76.5±14.48^{e}	67.97±2.44^{de}	52.65±7.51^{bc}	41.15±3.95^{ab}	38.55±5.53^{a}	43.51±7.40^{ab}	43.03±2.98^{ab}	49.44±1.17^{ab}
endo−2−Methyl bicyclo[3.3.1]nonane	1083.34372	0.85±0.12	–	–	–	–	–	–	1.53±0.16	–	–	–
cis−5−ethenyltetrahydro−α, α, 5−trimethyl−2−Furanmethanol	1088	90.90±3.28^{cd}	88.30±8.28^{cd}	103.91±8.67^{de}	93.83±18.50^{cde}	108.56±3.73^{e}	79.86±12.01^{bc}	65.34±8.57^{ab}	52.30±9.05^{a}	61.88±6.83^{a}	66.3±3.94^{ab}	66.69±8.19^{ab}
3,5−Octadien−2−one	1093	–	–	–	–	19.47±0.73	–	9.12±0.41	–	–	–	–
3−Cyclohexen−1−one, 3,5,5−trimethyl−	1097	1.56±0.25	–	–	–	–	–	–	–	–	–	–
2−Cyclohexen−1−one, 3,4,4−trimethyl−	1097	–	–	0.63±0.05	–	0.94±0.10	0.73±0.14	–	–	0.67±0.11	–	–
Linalool	1106	271.51±2.55^{de}	253.54±15.61^{de}	275.46±46.89^{e}	482.41±5.36^{g}	332.09±29.48^{f}	231.47±27.77^{cd}	182.61±19.81^{ab}	161.46±29.32^{a}	209.00±6.52^{bc}	201.65±8.40^{abc}	184.53±20.76^{ab}

3,7−dimethyl−1,5,7−Octatrien−3−ol	1106	70.30±1.19^{def}	63.29±5.05^{cd}	82.77±13.45^{f}	78.88±14.00^{ef}	112.34±3.17^{g}	67.20±11.20^{de}	51.62±2.36^{bc}	43.34±8.27^{ab}	29.66±4.87^{a}	41.49±1.24^{ab}	37.53±1.89^{a}
(E,E)−2,4−Octadienal	1113	–	–	–	–	–	–	–	3.16±0.45	–	3.53±0.07	–
2,6−dimethyl−Cyclohexanol	1114	28.45±0.56^{bc}	27.36±3.32^{bc}	43.77±7.89^{d}	31.94±3.72^{c}	32.85±5.38^{c}	23.78±4.75^{b}	13.27±2.32^{a}	11.37±1.90^{a}	13.43±1.89^{a}	11.54±0.61^{a}	16.37±0.01^{a}
Phenylethyl Alcohol	1116	22.40±0.32	19.74±1.41	–	34.19±5.41	32.15±0.47	–	–	12.63±2.50	–	13.73±1.58	–
2−ethyl−Hexanoic acid	1116.7	–	4.03±0.50	–	6.62±1.15	–	–	–	–	4.68±0.53	–	–
2,6,6−trimethyl−2−Cyclohexene−1−carboxaldehyde	1122.9	–	–	–	–	4.65±0.74	–	6.71±0.41	8.39±0.84	5.51±0.94	8.16±0.76	9.35±1.53
Isophorone	1124	2.69±0.28^{a}	2.79±0.48^{a}	4.72±0.86^{cd}	5.18±0.67^{d}	5.61±0.60^{d}	3.94±0.47^{bc}	2.52±0.09^{a}	2.67±0.45^{a}	2.84±0.52^{a}	2.67±0.21^{a}	3.72±0.45^{b}
(E,Z)−2,6−dimethyl−2,4,6−Octatriene	1131	–	–	1.99±0.22	–	–	–	–	–	–	–	1.59±0.12
E,E−2,6−Dimethyl−1,3,5,7−octatetraene	1132	2.73±0.18	1.91±0.15	2.7±0.07	–	2.64±0.37	1.17±0.18	–	–	–	0.92±0.06	–
3−Nonen−2−one	1136	1.37±0.22^{ab}	1.49±0.14^{abc}	1.73±0.23^{abc}	2.79±0.34^{d}	3.50±0.62^{e}	2.51±0.27^{d}	1.24±0.17^{a}	1.32±0.16^{a}	1.48±0.24^{abc}	1.96±0.18^{c}	1.89±0.14^{bc}

Benzyl nitrile	1143	–	–	–	–	3.57±0.37	2.35±0.31	2.43±0.36	2.60±0.09	1.22±0.04	–	1.67±0.28
(R,S)−5−Ethyl−6−methyl−3E−hepten−2−one	1143.9	6.47±1.25	–	–	–	–	–	–	–	–	–	–
1,2−dimethoxy−Benzene	1146	6.51±1.21^{a}	11.85±1.11^{de}	16.85±0.59^{f}	17.19±1.41^{f}	17.24±0.52^{f}	16.19±0.95^{f}	9.68±1.61^{bc}	8.28±1.34^{ab}	7.99±0.63^{ab}	10.65±0.19^{cd}	13.52±0.09^{e}
(E,E)−2,6−Nonadienal	1146	–	–	–	–	–	1.89±0.26	–	1.38±0.11	–	–	1.49±0.27
2,3,3−trimethyl−Bicyclo[2.2.1]heptan−2−ol	1148	5.41±0.61^{ef}	4.97±0.92^{cde}	5.39±0.21^{ef}	7.85±0.97^{g}	6.21±0.16^{f}	5.14±0.52^{de}	4.32±0.49^{bcd}	3.15±0.04^{a}	4.01±0.83^{abc}	5.94±0.12^{ef}	3.78±0.01^{ab}
1−Nonanol	1169.2	10.81±0.17^{cd}	8.38±0.42^{ab}	11.53±1.74^{d}	17.39±1.81^{e}	19.69±2.21^{f}	10.48±0.52^{cd}	8.98±0.36^{bc}	8.03±1.46^{ab}	6.94±0.48^{ab}	7.74±0.07^{ab}	6.53±0.09^{a}
3,4−Dimethoxytoluene	1172	–	–	–	–	–	–	2.03±0.29	–	–	3.09±0.13	4.14±0.84
DL−Menthol	1173	48.93±0.51^{ab}	58.07±1.32^{bc}	74.09±10.77^{d}	77.28±11.84^{d}	67.90±7.93^{cd}	45.88±9.17^{ab}	48.32±2.93^{ab}	46.46±5.97^{ab}	45.21±0.99^{ab}	39.29±2.84^{a}	176.36±15.6^{e}
Octanoic acid	1173	14.04±0.41^{c}	15.77±0.98^{c}	26.17±4.85^{de}	29.59±4.36^{ef}	32.27±2.50^{f}	23.99±1.71^{d}	4.60±0.34^{a}	7.59±1.22^{ab}	7.69±1.30^{ab}	9.55±1.81^{b}	15.06±2.67^{c}
(R)−4−methyl−1−(1−methylethyl)−3−Cyclohexen−1−ol	1175	13.83±0.27^{a}	19.14±1.47^{b}	22.41±3.93^{bc}	32.01±5.03^{d}	31.49±3.08^{d}	25.00±2.56^{c}	19.52±1.30^{b}	18.24±3.07^{ab}	21.57±1.50^{bc}	22.86±0.80^{bc}	21.79±1.69^{bc}

(3R,6S)−2,2,6−Trimethyl−6−vinyltetrahydro−2H−pyran−3−ol	1183	8.88±0.55^{c}	8.23±1.24^{abc}	12.01±2.03^{d}	14.64±2.63^{e}	11.63±1.26^{d}	8.25±0.38^{abc}	7.13±1.16^{abc}	6.01±0.71^{a}	8.44±0.70^{bc}	6.29±0.56^{ab}	7.52±0.01^{abc}
Methyl salicylate	1187	39.34±0.18	29.65±2.16	35.28±4.83	37.53±3.98	−	−	−	−	−	−	−
4−trimethyl−α,α−Benzenemethanol	1188	−	−	−	−	−	−	−	−	−	−	7.23±0.58
α−Terpineol	1190	102.34±0.95^{a}	136.06±8.53^{bc}	165.38±28.08^{d}	193.80±8.27^{e}	200.51±12.53^{e}	152.52±11.32^{bcd}	125.56±20.12^{ab}	103.74±18.79^{a}	152.97±13.65^{bcd}	133.27±8.64^{bc}	159.20±20.92^{cd}
Naphthalene	1190	2.48±0.16	3.40±0.20	4.29±0.63	−	7.94±0.61	−	−	−	4.14±0.18	−	−
1−ethyl−2,5−Pyrrolidinedione	1191	2.90±0.36	−	−	14.26±2.61	−	−	6.88±1.17	4.39±0.86	6.94±1.22	6.30±0.38	5.52±0.25
2,6,6−trimethyl−1,3−Cyclohexadiene−1−carboxaldehyde	1205	24.66±0.15^{d}	22.10±1.60^{c}	26.42±0.11^{de}	32.52±3.37^{f}	35.64±0.08^{g}	27.49±2.06^{e}	20.81±0.18^{c}	14.67±0.09^{a}	17.96±0.67^{b}	16.29±1.32^{ab}	16.06±0.77^{ab}
2,3−dihydro−Benzofuran	1219	6.05±0.18	5.11±0.56	5.99±0.93	7.64±1.31	8.01±1.3	4.69±0.09	3.74±0.58	2.98±0.37	4.18±0.48	−	−
2,6,6−trimethyl−1−Cyclohexene−1−carboxaldehyde	1220	24.58±0.27^{d}	17.79±1.31^{c}	19.28±1.60^{c}	26.38±0.49^{e}	27.02±1.39^{e}	13.92±1.17^{b}	10.2±0.25^{a}	8.32±1.44^{a}	9.82±0.26^{a}	9.07±0.27^{a}	9.93±1.29^{a}

(Z)−3,7−dimethyl−2,6−Octadien−1−ol	1229	13.34±0.56^{a}	15.85±1.18^{ab}	20.89±3.5^{c}	30.34±5.58^{d}	28.89±4.37^{d}	19.98±0.65^{bc}	12.41±1.81^{a}	12.58±2.31^{a}	16.28±0.63^{abc}	14.45±1.12^{a}	13.31±1.17^{a}
Geraniol	1258	43.95±1.12^{bcd}	48.22±3.67^{cd}	64.18±11.11^{e}	78.40±1.24^{f}	82.65±3.82^{f}	51.55±1.73^{d}	32.38±6.4^{a}	31.83±6.24^{a}	41.33±2.65^{abc}	35.81±2.48^{ab}	38.93±7.02^{abc}
Nonanoic acid	1272.1	23.46±1.49^{c}	22.45±1.56^{c}	41.20±3.75^{ef}	36.43±5.82^{e}	43.80±4.25^{f}	28.84±3.00^{d}	9.37±1.39^{a}	10.63±1.78^{ab}	14.64±2.14^{ab}	15.60±1.83^{b}	11.26±0.02^{ab}
6−Undecanone	1274	16.73±0.62^{de}	13.70±1.14^{bc}	16.81±2.70^{de}	14.53±0.10^{cd}	19.20±3.09^{e}	11.11±1.64^{b}	6.71±0.69^{a}	8.20±1.35^{a}	7.09±0.79^{a}	7.56±1.28^{a}	8.49±0.18^{a}
Indole	1294	−	−	−	−	−	−	−	−	−	1.26±0.09	−
1−Tridecene	1294	−	−	1.81±0.25	2.63±0.04	2.38±0.27	0.94±0.13	1.56±0.15	1.49±0.25	1.49±0.26	1.37±0.28	1.28±0.02
2,6,10,10−tetramethyl−1−Oxaspiro[4.5]dec−6−ene	1305.2	6.59±0.89	6.91±1.18	5.19±0.52	−	−	4.41±0.59	−	−	2.56±0.30	−	3.61±0.42
5,6,7,7a−tetrahydro−3,6−dimethyl−2(4H)−Benzofuranone	1314	6.23±0.89	−	−	−	−	−	−	−	−	−	−
1,2,3−Trimethoxybenzene	1317	−	−	2.33±0.32	−	3.47±0.22	2.52±0.16	2.88±0.44	2.14±0.30	0.92±0.11	2.68±0.24	1.90±0.20
2,6−dimethoxy−Phenol	1357	1.32±0.06	1.84±0.36	−	−	−	−	−	−	−	−	−

dihydro−5−pentyl−2(3H)−Furanone	1362	7.83±0.15^{f}	2.84±0.17^{a}	5.15±0.63^{d}	3.83±0.63^{bc}	9.06±0.17^{g}	6.00±0.60^{e}	4.38±0.50^{c}	3.08±0.54^{ab}	3.07±0.51^{ab}	5.79±0.36^{de}	3.60±0.26^{abc}
10−Undecenoic acid, methyl ester	1371	13.9±0.39	14.65±1.05	16.25±1.70	–	16.55±1.88	–	–	–	–	–	–
Propanoic acid, 2−methyl−,	1380	0.99±0.07^{a}	1.09±0.03^{a}	3.45±0.50^{bc}	4.33±0.05^{cd}	5.16±0.73^{d}	2.87±0.43^{b}	1.54±0.30^{a}	9.35±1.48^{e}	3.96±0.54^{c}	4.34±0.17^{cd}	3.98±0.43^{c}
(E)−1−(2,6,6−trimethyl−1,3−cyclohexadien−1−yl)−2−Buten−1−one	1388	3.38±0.01^{ab}	4.05±0.39^{bc}	5.4±0.48^{d}	5.69±0.69^{d}	6.73±0.80^{e}	4.62±0.07^{c}	2.99±0.01^{a}	2.79±0.25^{a}	3.10±0.26^{a}	2.82±0.01^{a}	2.93±0.36^{a}
1−Tetradecene	1392	–	–	0.62±0.11	–	–	–	2.99±0.07	–	1.20±0.24	–	1.98±0.32
6,10−dimethyl−2−Undecanone	1407	3.55±0.02	3.94±0.33	4.05±0.34	–	6.08±0.39	5.47±0.68	–	4.02±0.26	4.20±0.05	–	–
[3R−(3α,3aβ,7β,8aα)]−2,3,4,7,8,8a−hexahydro−3,6,8,8−tetramethyl−1H−3a,7−Methanoazulene	1408	–	–	17.11±2.37	20.25±3.83	16.42±1.83	20.17±3.77	6.75±0.57	6.85±1.16	6.44±0.97	10.93±0.46	18.69±3.47
Dodecanal	1412	–	1.42±0.20	–	4.66±0.65	1.25±0.20	1.18±0.09	0.84±0.14	0.98±0.09	–	2.45±0.14	1.27±0.02

Longifolene	1412.6	–	–	–	–	4.11±0.66	2.94±0.54	4.58±0.39	3.23±0.43	–	3.40±0.71	1.43±0.16
α−Ionone	1421	15.43±2.62^{b}	15.14±0.62^{b}	15.13±1.86^{b}	22.50±4.34^{c}	20.08±1.97^{c}	12.99±0.56^{ab}	10.08±0.28^{a}	11.30±1.48^{a}	10.96±0.53^{a}	13.04±2.33^{ab}	15.44±1.88^{b}
6−Methyl−6−(5−methylfuran−2−yl)heptan−2−one	1426	–	–	3.79±0.44	33.09±5.04	–	2.83±0.31	–	–	–	–	–
4−(2,2,6−trimethyl−7−oxabicyclo[4.1.0]hept−1−yl)−3−Buten−2−one	1428	–	–	8.27±1.49	–	–	–	–	–	–	2.53±0.35	–
Ionone	1429	–	–	23.66±0.28	–	–	19.49±1.88	10.24±0.92	4.26±0.54	–	4.04±0.52	–
4−(2,6,6−trimethyl−1−cyclohexen−1−yl)−2−Butanone	1433	4.48±0.36	–	2.48±0.32	2.75±0.24	–	–	–	–	–	–	3.73±0.25
6,10−dimethyl−5,9−Undecadien−2−one	1434	34.61±6.73^{d}	29.03±0.59^{c}	31.08±3.02^{cd}	16.66±2.97^{ab}	32.01±3.25^{cd}	20.95±0.92^{b}	11.81±0.46^{a}	13.46±1.74^{a}	12.94±0.14^{a}	14.56±0.89^{a}	14.19±2.36^{a}
4−(2,6,6−Trimethylcyclohexa−1,3−dienyl)but−3−en−2−one	1440	8.04±0.23^{a}	13.17±0.81^{b}	17.77±2.81^{c}	23.54±1.55^{d}	26.62±2.64^{e}	19.55±0.75^{c}	11.73±0.86^{b}	11.58±1.50^{b}	13.08±0.23^{b}	11.62±0.47^{b}	13.19±2.58^{b}

trans−β−Ionone	1457	61.66±2.27^{c}	59.18±2.87^{c}	63.9±9.36^{c}	56.33±5.05^{c}	75.95±5.35^{d}	47.80±1.19^{b}	35.30±2.34^{a}	28.51±3.80^{a}	32.33±0.55^{a}	28.8±1.16^{a}	31.45±5.12^{a}
1−Dodecanol	1457	3.56±0.06^{a}	5.59±0.20^{b}	6.27±0.75^{bc}	5.68±0.74^{b}	9.00±0.62^{d}	7.30±0.22^{c}	5.71±0.46^{b}	5.73±1.13^{b}	7.46±0.68^{c}	6.96±0.93^{c}	7.22±0.24^{c}
(+)−Valencene	1491	4.85±0.33	4.41±0.29	5.4±0.91	–	4.94±0.35	2.21±0.23	2.57±0.09	2.00±0.32	–	–	–
cis−hexahydro−8a−methyl−1,8(2H,5H)−Naphthalenedione	1517	28.42±0.95	–	–	–	–	22.57±0.2	–	–	16.95±0.67	–	–
Butylated Hydroxytoluene	1517.5	1.39±0.13	1.50±0.08	1.66±0.30	1.03±0.02	0.99±0.09	0.66±0.13	–	–	–	–	–
(R)−5,6,7,7a−tetrahydro−4,4,7a−trimethyl−2(4H)−Benzofuranone,	1525	31.72±1.51^{ab}	33.55±3.10^{abc}	43.40±6.44^{bc}	72.09±14.23^{d}	72.4±10.57^{d}	45.21±6.16^{c}	33.06±1.47^{abc}	30.34±5.95^{a}	44.20±5.88^{bc}	34.16±1.14^{abc}	33.48±1.59^{abc}
(E)−3,7,11−trimethyl−1,6,10−Dodecatrien−3−ol	1551	4.31±0.14	7.80±0.22	–	–	–	7.56±0.24	–	–	4.03±0.20	–	4.24±0.15
2,4−Di−tert−butylphenol	1555	16.86±1.65^{a}	19.54±1.14^{ab}	21.46±3.21^{abc}	19.50±3.05^{ab}	35.94±2.87^{d}	26.11±0.39^{c}	22.37±1.31^{bc}	19.52±2.73^{ab}	24.38±3.57^{bc}	24.94±4.67^{c}	24.68±3.04^{bc}
n−Nonylcyclohexane	1556	–	–	–	–	–	–	–	–	4.66±0.29	6.94±0.95	6.10±0.88

Methanone, dicyclohexyl−	1576	18.32±0.71^{bcd}	18.43±1.31^{bcd}	21.86±3.22^{d}	21.84±4.21^{d}	28.70±3.46^{e}	19.76±0.95^{bcd}	16.31±2.43^{abc}	12.81±2.04^{a}	18.14±2.48^{bcd}	15.45±0.71^{ab}	20.52±3.81^{cd}
2,2,4−Trimethyl−1,3−pentanediol diisobutyrate	1587.5	7.82±0.15^{b}	15.79±0.66^{g}	16.20±0.46^{g}	10.78±0.20^{de}	9.14±0.68^{bc}	20.42±0.46^{h}	5.73±0.87^{a}	10.15±1.67^{cd}	11.94±0.60^{e}	13.91±0.21^{f}	13.90±1.77^{f}
Cedrol	1607.9	–	11.23±0.02	18.63±1.21	7.36±1.05	17.59±1.29	12.45±0.08	15.94±0.56	30.98±4.02	30.57±2.25	37.09±0.57	31.47±4.95
Tetradecanal	1614	–	–	–	1.63±0.05	–	–	5.98±0.23	–	–	–	7.23±1.46
Benzophenone	1664	–	–	14.89±2.63	3.83±0.12	9.30±0.49	11.12±1.08	–	8.18±0.94	10.74±0.29	7.49±1.23	7.25±0.74
2,2',5,5'−tetramethyl−1,1'−Biphenyl	1668.5	–	–	–	–	–	–	1.56±0.09	1.22±0.18	–	1.22±0.02	1.39±0.16
2−hexyl−1−Decanol	1790	5.40±0.18	–	–	–	–	–	–	–	6.05±0.77	9.29±0.05	6.56±1.18
3,4−diethyl−1,1'−Biphenyl	1792	–	11.24±0.26	–	–	–	28.4±1.82	–	12.51±1.53	–	–	10.7±0.49
Neophytadiene	1840	21.12±0.02^{e}	22.90±2.62^{ef}	25.74±5.10^{f}	16.80±1.99^{cd}	25.51±0.77^{f}	19.01±2.47^{de}	13.37±2.20^{bc}	13.39±2.31^{bc}	16.89±0.72^{cd}	11.20±0.05^{ab}	8.99±1.41^{a}
Dodecylcyclohexane	1864	2.73±0.36^{a}	3.48±0.13^{ab}	8.83±1.48^{e}	6.67±0.73^{d}	4.66±0.41^{bc}	6.84±0.55^{d}	5.97±0.20^{cd}	4.67±0.68^{bc}	4.80±0.75^{bc}	5.84±0.95^{cd}	4.01±0.55^{b}
Phytol	2045	16.50±1.15^{de}	24.40±2.06^{f}	23.47±1.53^{f}	14.73±2.26^{cd}	16.69±1.21^{de}	18.51±3.07^{e}	14.36±2.81^{cd}	11.28±2.02^{bc}	13.83±0.87^{cd}	8.48±1.24^{ab}	7.63±0.99^{a}
Decanoic acid, methyl ester	1282	–	–	–	16.38±1.25	–	14.87±1.95	10.06±0.38	9.18±0.42	–	–	–

n−Hexadecanoic acid	1968	–	–	–	–	40.59±4.43	–	–	–	–	–	–
1−Decanol	1258	–	–	–	–	9.67±0.92	5.48±0.3	–	4.07±0.29	–	–	–
Sulfurous acid, butyl tetradecyl ester	2434	3.34±0.56	2.15±0.19	–	–	2.22±0.20	1.87±0.08	–	–	–	1.25±0.15	1.33±0.12

[a] RI =Retention index of aroma compounds on HP−5MS UI Column; RI were obtained from NIST Chemistry WebBook.(https://webbook.nist.gov/chemistry/name−ser/)

[b] Concentrations of aroma compounds in T0, T1, T2,T3, T4, T5, T6, T7, T8, T9 and T10 were represented RAPT samples from eleven different aging years, respectively; values with different letters (a−h) in a row are significantly using Duncan's multiple comparison tests ($p < 0.05$) .

[c] All the above aroma compounds were identified by MS and RI values, except. Phytol, Decanoic acid, methyl ester, n−Hexadecanoic acid , 1−Decanol and Sulfurous acid, butyl tetradecyl ester is identified by MS

Table S2

Key volatile compounds identified in RAPT samples from different years of storage

Volatile compounds	Class	Odor	Threshold(μg/kg)	OAV											ACI(%)
				T0	T1	T2	T3	T4	T5	T6	T7	T8	T9	T10	
DL−Menthol	Woody	mint−like,woody	1000	0.5	0.6	0.7	0.8	0.7	0.5	0.5	0.5	0.5	0.4	1.8	<0

Linalool	Floral&fruity	Citrus−like, flowery	0.6	4525.1	4225.6	4591.1	8040.2	5534.9	3857.9	3043.5	2691.1	3483.4	3360.8	3075.5	0.72~8.28
α−Terpineol	Floral	flowery	4.08	250.8	333.5	405.3	475	491.5	373.8	307.7	254.3	374.9	326.7	390.2	0.06~0.63
Geraniol	Floral&fruity	Rose−like, citrus−like	3.2	137.3	150.7	200.6	245	258.3	161.1	101.2	99.5	129.1	111.9	121.7	0.03~0.25
trans−Linalool oxide (furanoid)	Floral	floral	3000	0.2	0.1	0.2	0.3	0.2	0.2	0.1	0.1	0.1	0.1	0.2	<0
trans−β−Ionone	Floral	Flowery, violet−like	0.007	88081.6	84544.4	91283.8	80478.2	108493	68278.6	50430.3	40730.3	46186	41143.2	44931.8	14.41~85.82
D−Limonene	Fruity	Citrus−like, carrot−like	13	34.1	52.5	57.6	21.4	57.3	44.1	35	42.6	40.8	53.4	33.8	0.01~0.09
Benzaldehyde	Fruity	Cherry−like	320	0.5	0.6	1.1	0.8	1.5	0.8	0.6	0.6	0.7	0.7	0.8	<0
1H−Pyrrole−2−carboxaldehyde, 1−ethyl−	Roasted	Bread−like, roasty	65000	<0.1	<0.1	<0.1	<0.1	<0.1	<0.1	<0.1	<0.1	<0.1	<0.1	<0.1	<0

Octanoic acid	Cheese flavor	Carrot−like, musty Cheese, fat	190	0.7	0.8	1.4	1.6	1.7	1.3	0.2	0.4	0.4	0.5	0.8	<0
α−Ionone	Floral	Flowery, violet−like	0.4	385.8	378.5	378.1	562.5	501.9	324.9	251.9	282.4	274.1	326	386	0.06~0.62
Benzeneacetaldehyde	Floral	Lilac and hyacinth−like	4	29.8	24.9	30	56.7	56.1	29.1	30.6	30.6	48.1	35.3	42.9	0~0.06
2,6,6−trimethyl−1,3−Cyclohexadiene−1−carboxaldehyde	Woody	Woody, herbal	3	82.2	73.7	88.1	108.4	118.8	91.6	69.4	48.9	59.9	54.3	53.5	0.01~0.12
(E,E)−2,4−Heptadienal	Floral	Fatty, flowery	0.032	3598.6	3248.6	3338.5	4774.2	5892	3473.1	4117.8	3857.1	4501.8	4416.6	4351.2	0.55~7.76
1,2−dimethoxy−Benzene	Stale	Sweet and Musty	3.17	20.5	37.4	53.2	54.2	54.4	51.1	30.5	26.1	25.2	33.6	42.7	0.01~0.06
(Z)−3,7−dimethyl−2,6−Octadien−1−ol	Floral&fruity	Citrus−like,Floral	49	2.7	3.2	4.3	6.2	5.9	4.1	2.5	2.6	3.3	2.9	2.7	0~0.01

6−methyl−5−Hepten−2−one	Fruity	Citrus−like, apple−like	50	3.9	3	2.6	3.3	4.4	3.5	2.2	2	1.9	2.7	2.6	0~0.01
1−Octanol	Floral&fruity	Rose−like or lemon−like	0.8	255.1	317.3	346	620.8	497.9	336.4	106.6	95.2	158.4	126.8	131.8	0.06~0.64
Nonanoic acid	Stale	Moldy, pungent	26	9	8.6	15.8	14	16.8	11.1	3.6	4.1	5.6	6	4.3	0~0.01
2,6,6−trimethyl−1−Cyclohexene−1−carboxaldehyde	−	−	5	49.2	35.6	38.6	52.8	54	27.8	20.4	16.6	19.6	18.1	19.9	0.01~0.05
1−Octen−3−ol	Green	Mushroom−like, green	45	21.3	11.5	5.9	5.1	8.2	4.8	2.6	2.7	2.8	3.8	3.4	0~0.01
1−(1H−pyrrol−2−yl)−Ethanone	Nutty	Nutty, musty	3.4	41.2	39.3	39	75.9	68	49	34.8	35.3	38.1	36.4	29.2	0.01~0.08
Octanal	Fruity	Citrus−like, soapy	0.0455	1600.9	1455.3	1320.7	1376.8	4131.4	2432.5	1534.7	1365	1880.6	1836.4	1632.4	0.25~3.52
1−Dodecanol	Fatty	Fatty, soapy	6.1	5.8	9.2	10.3	9.3	14.8	12	9.4	9.4	12.2	11.4	11.8	0~0.02

1−Nonanol	Floral	rose−like	11	9.8	7.6	10.5	15.8	17.9	9.5	8.2	7.3	6.3	7	5.9	0~0.02
Heptanal	Fatty	Citrus−like, fatty	9.7	4.7	4.7	8	6.9	9	7.3	7.2	3.5	7.8	5.9	5.3	0~0.01
Isophorone	Woody	Woody	3000	<0.1	<0.1	<0.1	<0.1	<0.1	<0.1	<0.1	<0.1	<0.1	<0.1	<0.1	<0
Dihydro−5−pentyl−2(3H)−Furanone	Cheese flavor	Coconut, creamy, waxy with fatty milky notes	4.5	17.4	6.3	11.5	8.5	20.1	13.3	9.7	6.8	6.8	12.9	8	0~0.02
(E)−1−(2,6,6−trimethyl−1,3−cyclohexadien−1−yl)−,2−Buten−1−one	Floral&fruity	apple−like, rose−like	1000	<0.1	<0.1	0.1	0.1	0.1	<0.1	<0.1	<0.1	<0.1	<0.1	<0.1	<0
2−Heptanone	Fruity	cabbage−like	70	0.6	0.6	0.4	0.5	0.8	0.7	0.4	0.3	0.4	0.6	0.3	<0
3−methyl−Butanoic acid	Cheese flavor	Cheese−like	10000	<0.1	<0.1	<0.1	<0.1	<0.1	<0.1	<0.1	<0.1	<0.1	<0.1	<0.1	<0
1−Hexanol	Green	Green, grassy	40	1.4	1.4	0.8	1	1.2	0.8	0.6	0.5	0.8	0.4	0.6	<0

p−Xylene	Chemical	Plastic, green,	60	0.3	0.3	0.3	1	0.7	0.3	0.4	0.4	0.5	0.4	0.4	<0
2−methyl−Butanoic acid	Cheese flavor	Butter, cheese−like	15	3.4	3	1.6	2.2	2.1	1.7	0.4	0.7	0.4	0.9	1	<0
α−Pinene	Woody	Fresh, woody	48	0.1	0.1	0.1	0.1	0.2	0.2	0.2	0.2	0.2	0.2	0.2	<0
(Z)−3−Hexen−1−ol	Green	Grass	0.0002	151243.8	145866.1	ND	ND	ND	ND	ND	ND	ND	ND	ND	0~40.33
1−ethyl−1H−Pyrrole	Roasted	Burnt, roasted	3	4.6	3.2	ND	ND	ND	ND	ND	ND	ND	ND	ND	<0
Methyl salicylate	Green	wintergreen	10	39.3	29.7	35.3	37.5	ND	ND	ND	ND	ND	ND	ND	0~0.04
Phenylethyl Alcohol	Floral&fruity	Rose−like	9	24.9	21.9	29.4	38	35.7	19.1	ND	14	7	15.3	ND	0~0.04
β−Myrcene	Woody	Pepper−like,carrot−like	0.75	ND	239.3	ND	ND	ND	280.2	ND	ND	ND	215.1	171.3	0~0.41
Tetradecanal	Chemical	Alkane	60	ND	ND	ND	0.3	ND	ND	1	ND	ND	ND	1.2	<0
1−Decanol	Floral&fruity	orange−like, floral	23	ND	ND	ND	ND	4.2	2.4	ND	1.8	ND	ND	ND	<0
Naphthalene	Chemical	pungent dry tarry	50	0.5	0.7	0.9	ND	1.6	ND	ND	ND	0.8	ND	ND	<0

2,3−dihydro−Benzofuran	Chemical	Tar−like, pungent	48	1.3	1.1	1.2	1.6	1.7	1	0.9	0.6	0.9	ND	ND	<0
1−Oxaspiro[4.5]dec−6−ene, 2,6,10,10−tetramethyl−	Woody	Herbal	0.0002	329594.6	345510	259316.6	ND	ND	220607.8	ND	ND	128021.3	ND	180540.2	0~76.48
Benzyl alcohol	Floral&fruity	Rose−like, cherry−like	5500	<0.1	ND	ND	<0.1	ND	ND	ND	ND	ND	ND	ND	<0
Ionone	Floral	Flowery, violet−like	1500	ND	ND	0.2	ND	ND	0.1	0.1	<0.1	ND	<0.1	ND	<0
(E)−2−Octenal	Green	Green	3	ND	ND	ND	ND	ND	ND	ND	7	ND	9.6	ND	0~0.02
2,6−dimethoxy−Phenol	Chemical	phenolic, smoky, woody	400	v	<0.1	ND	ND	ND	ND	ND	ND	ND	ND	ND	<0
(E,E)−2,6−Nonadienal	Green	cucumber−like, grren	1	ND	ND	ND	ND	ND	18.9	ND	13.8	ND	ND	14.9	0~0.03
Dodecanal	Green	Green,soapy,citrus−like	1.07	ND	13.3	ND	43.5	11.7	11.1	7.8	9.2	ND	22.9	11.9	0~0.04

Indole	Floral	Floral, animal−like	500	0.1	ND	<0.1	ND	ND	ND	ND	ND	<0.1	<0.1	ND	<0
Hexanoic acid, methyl ester	Fruity	Pine apple−like	10	ND	ND	ND	ND	1	ND	0.4	2.4	0.5	ND	ND	<0
2−Octanone	Woody	Woody, Herbal	50	0.1	ND	ND	ND	ND	0.3	ND	ND	ND	ND	ND	<0
2−butoxy−Ethanol	−	−	2600	ND	<0.1	ND	ND	<0.1	ND	<0.1	<0.1	<0.1	<0.1	<0.1	<0
Hexanal	Green	Green	9	ND	0.5	6.7	ND	ND	1.5	0.5	0.6	0.6	0.7	0.7	<0
1,2,3−Trimethoxybenzene	Stale	Stale and Musty	0.75	ND	ND	31.1	ND	46.3	33.6	38.4	28.5	12.3	35.7	25.3	0~0.07

[a] OT = Odor thresholds

[b] ND, the compounds was not detected

[c] All the odor thresholds were obtained from previous research [9, 27, 36 − 39].

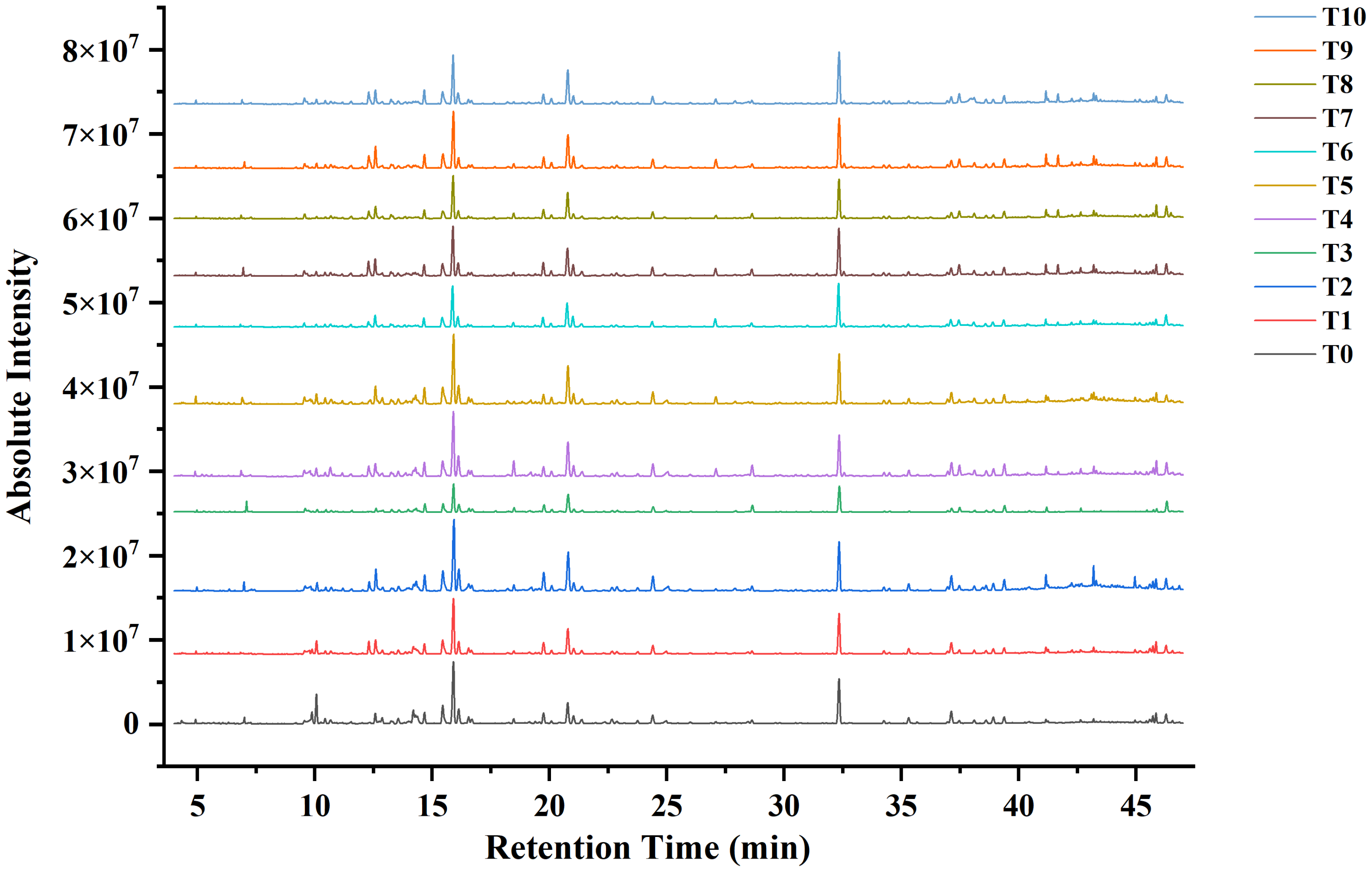

Absolute Intensity
8×10^7
7×10^7
6×10^7
5×10^7
4×10^7
3×10^7
2×10^7
1×10^7
0
5
10
15
20
25
30
35
40
45
Retention Time (min)
T10
T9
T8
T7
T6
T5
T4
T3
T2
T1
T0

Figure S1 Total ion chromatograms (TIC) of aroma compounds in RAPT

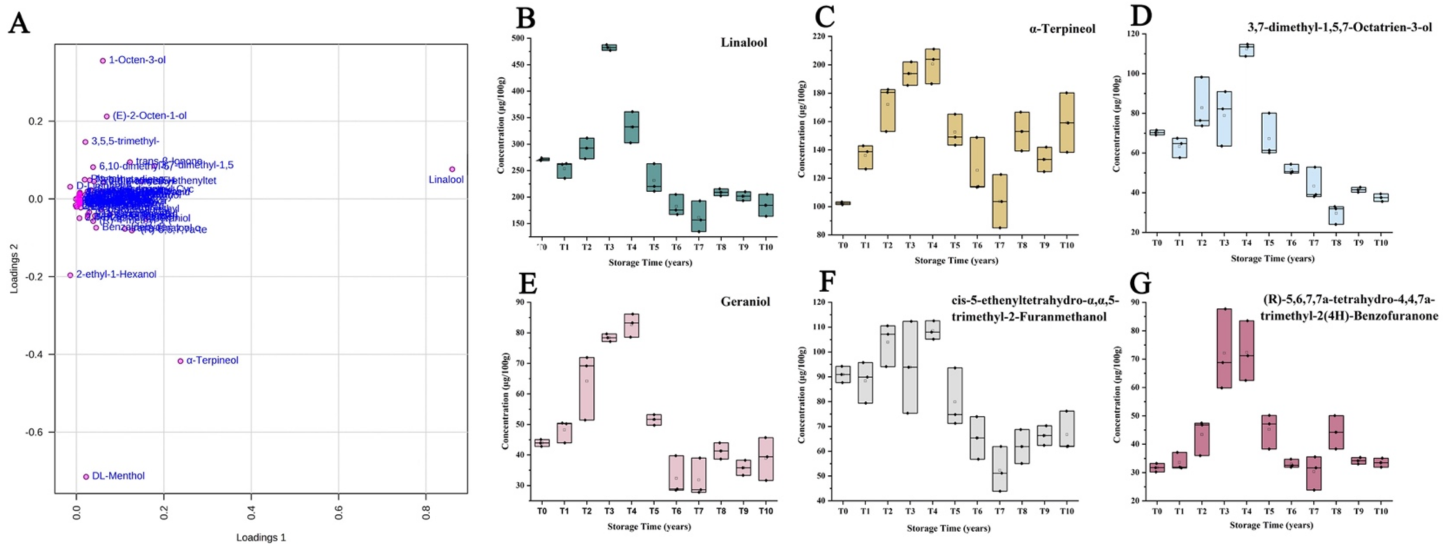

Figure S2 Multivariate statistical analysis of volatile compounds and during RAPT storage (a) loadings plot (b−g) Comparison of the accurate contents of differential compounds screened by loadings plot

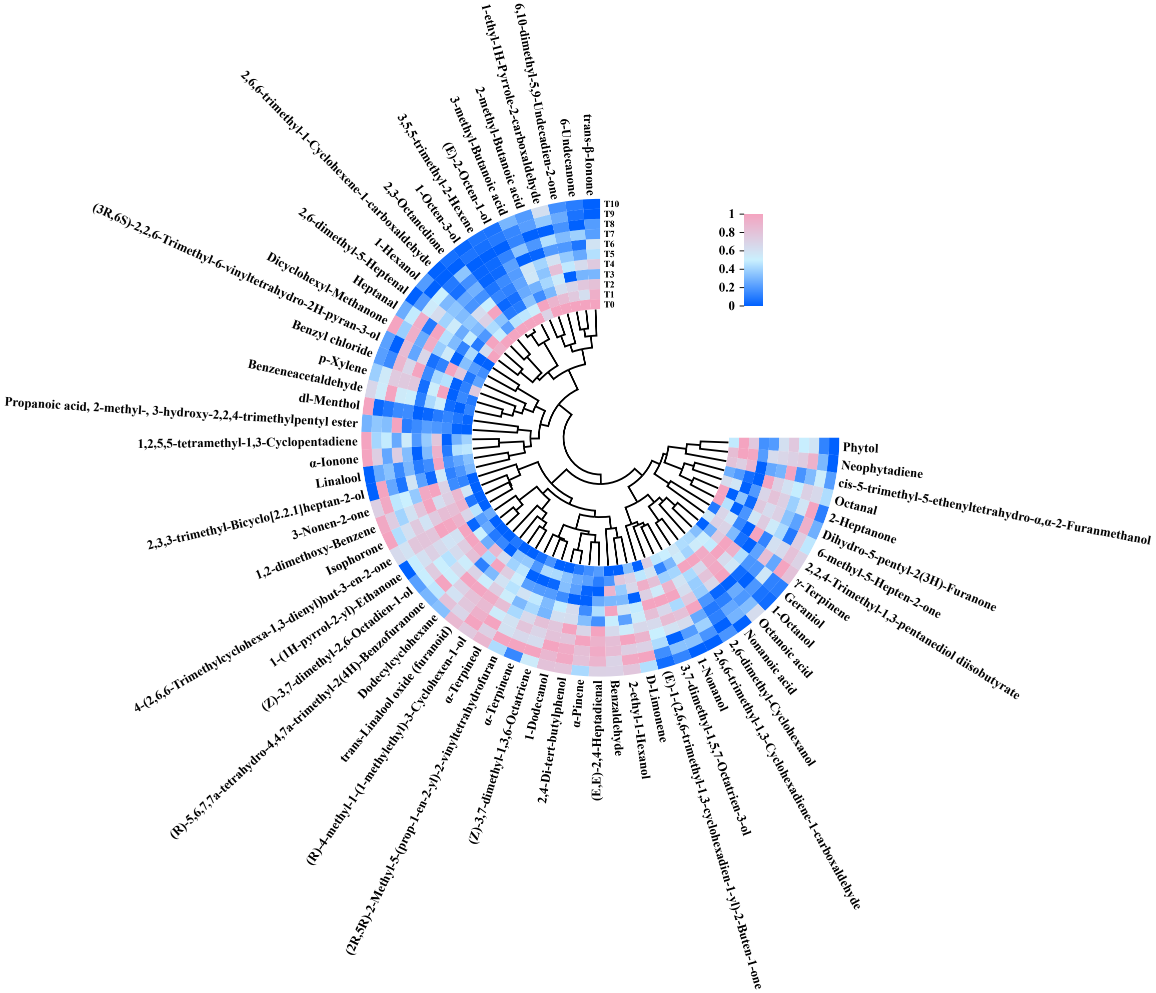

trans-β-Ionone
6-Undecanone
6,10-dimethyl-5,9-Undecadien-2-one
1-ethyl-1H-Pyrrole-2-carboxaldehyde
2-methyl-Butanoic acid
3-methyl-Butanoic acid
(E)-2-Octen-1-ol
3,5,5-trimethyl-2-Hexene
1-Octen-3-ol
2,3-Octanedione
2,6,6-trimethyl-1-Cyclohexene-1-carboxaldehyde
1-Hexanol
2,6-dimethyl-5-Heptenal
Heptanal
Dicyclohexyl-Methanone
(3R,6S)-2,2,6-Trimethyl-6-vinyltetrahydro-2H-pyran-3-ol
Benzyl chloride
p-Xylene
Benzeneacetaldehyde
dl-Menthol
Propanoic acid, 2-methyl-, 3-hydroxy-2,2,4-trimethylpentyl ester
1,2,5,5-tetramethyl-1,3-Cyclopentadiene
α-Ionone
Linalool
2,3,3-trimethyl-Bicyclo[2.2.1]heptan-2-ol
3-Nonen-2-one
1,2-dimethoxy-Benzene
Isophorone
4-(2,6,6-Trimethylcyclohexa-1,3-dienyl)but-3-en-2-one
1-(1H-pyrrol-2-yl)-Ethanone
(Z)-3,7-dimethyl-2,6-Octadien-1-ol
(R)-5,6,7,7a-tetrahydro-4,4,7a-trimethyl-2(4H)-Benzofuranone
Dodecylcyclohexane
trans-Linalool oxide (furanoid)
(R)-4-methyl-1-(1-methylethyl)-3-Cyclohexen-1-ol
α-Terpineol
(2R,5R)-2-Methyl-5-(prop-1-en-2-yl)-2-vinyltetrahydrofuran
α-Terpinene
(Z)-3,7-dimethyl-1,3,6-Octatriene
1-Dodecanol
2,4-Di-tert-butylphenol
α-Pinene
(E,E)-2,4-Heptadienal
Benzaldehyde
2-ethyl-1-Hexanol
D-Limonene
(E)-1-(2,6,6-trimethyl-1,3-cyclohexadien-1-yl)-2-Buten-1-one
3,7-dimethyl-1,5,7-Octatrien-3-ol
1-Nonanol
2,6,6-trimethyl-1,3-Cyclohexadiene-1-carboxaldehyde
2,6-dimethyl-Cyclohexanol
Nonanoic acid
Octanoic acid
1-Octanol
Geraniol
γ-Terpinene
2,2,4-Trimethyl-1,3-pentanediol diisobutyrate
6-methyl-5-Hepten-2-one
Dihydro-5-pentyl-2(3H)-Furanone
2-Heptanone
Octanal
cis-5-trimethyl-5-ethenyltetrahydro-α,α-2-Furanmethanol
Neophytadiene
Phytol
T10
T9
T8
T7
T6
T5
T4
T3
T2
T1
T0
1
0.8
0.6
0.4
0.2
0

Figure S3 Heat map of differential volatile compounds in the samples of RAPT at various storage time points (Relative content)
